# Precision‐cut lung slices: A powerful ex vivo model to investigate respiratory infectious diseases

**DOI:** 10.1111/mmi.14817

**Published:** 2021-10-31

**Authors:** Flávia Viana, Cecilia M. O’Kane, Gunnar N. Schroeder

**Affiliations:** ^1^ Wellcome‐Wolfson Institute for Experimental Medicine School of Medicine, Dentistry and Biomedical Sciences Queen's University Belfast Belfast UK

**Keywords:** ex vivo, infection, organotypic models, precision‐cut lung slices, respiratory infections

## Abstract

Respiratory infections are a leading cause of mortality worldwide. Most of the research on the underlying disease mechanisms is based on cell culture, organoid, or surrogate animal models. Although these provide important insights, they have limitations. Cell culture models fail to recapitulate cellular interactions in the lung and animal models often do not permit high‐throughput analysis of drugs or pathogen isolates; hence, there is a need for improved, scalable models. Precision‐cut lung slices (PCLS), small, uniform tissue slices generated from animal or human lungs are increasingly recognized and employed as an ex vivo organotypic model. PCLS retain remarkable cellular complexity and the architecture of the lung, providing a platform to investigate respiratory pathogens in a near‐native environment. Here, we review the generation and features of PCLS, their use to investigate the pathogenesis of viral and bacterial pathogens, and highlight their potential to advance respiratory infection research in the future.

## Graphical abstract

Precision‐cut lung slices are organotypic ex vivo models that can be used to unveil cellular and molecular mechanisms underlying host‐pathogen interactions in a nearly native environment.

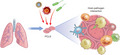

## 1 INTRODUCTION

Lower respiratory tract infections such as pneumonia are among the 10 most common causes of death worldwide and are a significant burden for healthcare systems (Gibson et al., 2013; World Health Organization, 2020). The swift onset of the SARS‐CoV‐2 pandemic further highlights that new respiratory pathogens can emerge rapidly. Hence, there is an urgent need to accelerate fundamental research into respiratory infection biology and to develop technologies that allow for its translation into optimized diagnosis and treatment strategies.

To date, fundamental respiratory research has mostly relied on cell culture and surrogate animal models to investigate the mechanisms underlying host‐pathogen interactions and different pathologies. Two‐dimensional (2D) cell culture has been the most employed model as it is cheap and easy‐to‐implement, but it has limitations. It predominantly relies on immortalized cell lines that often display significantly altered metabolism and gene expression profiles compared to primary cells (Sanderson, 2011). The use of primary cells can circumvent some of these drawbacks, as these retain more morphological and physiological characteristics of the source tissue. However, primary cells can only be retrieved in low numbers, expanded for a finite time, display limited differentiation capacity and, as the other 2D models, still lack the cellular heterogeneity, intercellular interactions, and complex structural organization of tissues. More complex three‐dimensional (3D) cell culture models address some of these limitations. For example, primary airway epithelial cells cultured at air liquid interface (ALI) develop into a differentiated, polarized airway‐like epithelium (Cao et al., 2020; Zscheppang et al., 2018). Stem/progenitor cells embedded in a matrix can self‐organize into complex lung tissue‐like organoids (Hiemstra et al., 2019; Nikolić & Rawlins, 2017). These models recapitulate many aspects of the airway epithelium, as the heterogeneous epithelial cell composition including mucus‐producing goblet and beating ciliated cells, and some degree of 3D organization. Other cells, for example macrophages, can be added to these 3D models, allowing the investigation of cellular crosstalk and immune mechanisms (Hiemstra et al., 2019). Integration with microfluidic “organ‐on‐a‐chip” systems, which continuously supply nutrients and remove waste, further enhances their potential to mimic in vivo conditions (Gkatzis et al., 2018; Hiemstra et al., 2019). However, current models are still unable to recapitulate the true cellular richness and spatial complexity of the lung.

Surrogate animal models allow experimentation under physiological conditions in a living organism and provide invaluable knowledge about the mechanisms underlying respiratory infectious diseases. Mice are the most frequently used model, due to the large range of genetically modified strains and tools that exist to study them. Guinea pigs reflect human airway pathology better than mice (Ressmeyer et al., 2006) and were the first model used to study tuberculosis and diphtheria. Although there are less tools available to study these, guinea pigs remain important surrogate models for *Legionella pneumophila, Brucella* spp., and several viruses (Padilla‐Carlin et al., 2008). Pigs have been increasingly employed since porcine airway anatomy, physiology and more than 80% of immune parameters closely resemble that of humans (Meurens et al., 2012). In contrast, mice only share 10% of immune parameters. Still, despite best efforts, significant genetic, anatomical, and physiological differences often hinder the direct translation of animal research to humans (Bonniaud et al., 2018; Williams & Roman, 2016).

To bridge this gap, ex vivo human lung tissue explants (HLTEs), which are usually obtained from resected tissue samples from patients undergoing surgery for example, for lung cancer, have been used for modeling infection with *L. pneumophila* (Jäger et al., 2014), *Chlamydophila pneumoniae* (Rupp et al., 2004), *Streptococcus pneumoniae* (Xu et al., 2008), and *Haemophilus influenzae* (Drömann et al., 2010). As usually only small areas of tissue are resected at one time, the number of HLTEs generated is small, limiting the number of experimental conditions that can be tested. Moreover, while tumor tissue itself is avoided, HLTEs are typically derived from a diseased lung environment.

Low availability of human tissue, the high cost of infrastructure and maintenance of laboratory animals, and substantial efforts made in recent years to implement the 3Rs principles, Refinement, Reduction, and Replacement for animal experimentation (European Parliament and Council directive 2010/63/EU, 2010; Hubrecht & Carter, 2019) led to the development and increased use of precision‐cut tissue slices (PCTS) as a 3D organotypic ex vivo tissue model. PCTS are clearly defined tissue sections of uniform thickness generated from a single piece of tissue. They can be generated in large numbers from diverse organs including the lung (precision‐cut lung slices—PCLS), from different animals and also from live or cadaveric human tissue and organs donated for research. This enables testing of a wide range of variables in replicate, reduces the amount of source tissue or animals needed and is therefore also significantly more cost‐effective when compared to in vivo work.

Here, we will describe the generation and characteristics of PCLS, review their use for studying human respiratory pathogens so far and discuss their potential for advancing respiratory infectious disease research and antimicrobial drug discovery.

## 2 GENERATION OF PCLS

Tissue slices have been used in research since the 1920's. However, it was the development of precise vibrating microtomes that enabled PCTS in 1980, increasing the homogeneity and reproducibility of the generated slices (Krumdieck et al., 1980). It took seven more years until the introduction of an agar infusion method provided enough structural support to the soft and fragile honeycomb architecture of lung tissue, making it amenable for slicing (Placke & Fisher, 1987). This method was further developed and first applied to human lung tissue in 1994 (Fisher et al., 1994). Various adaptations have been made since then, and detailed step‐by‐step protocols for generating PCLS from human and mouse tissue are published (Gerckens et al., 2019; Wu et al., 2019).

**FIGURE 1:**
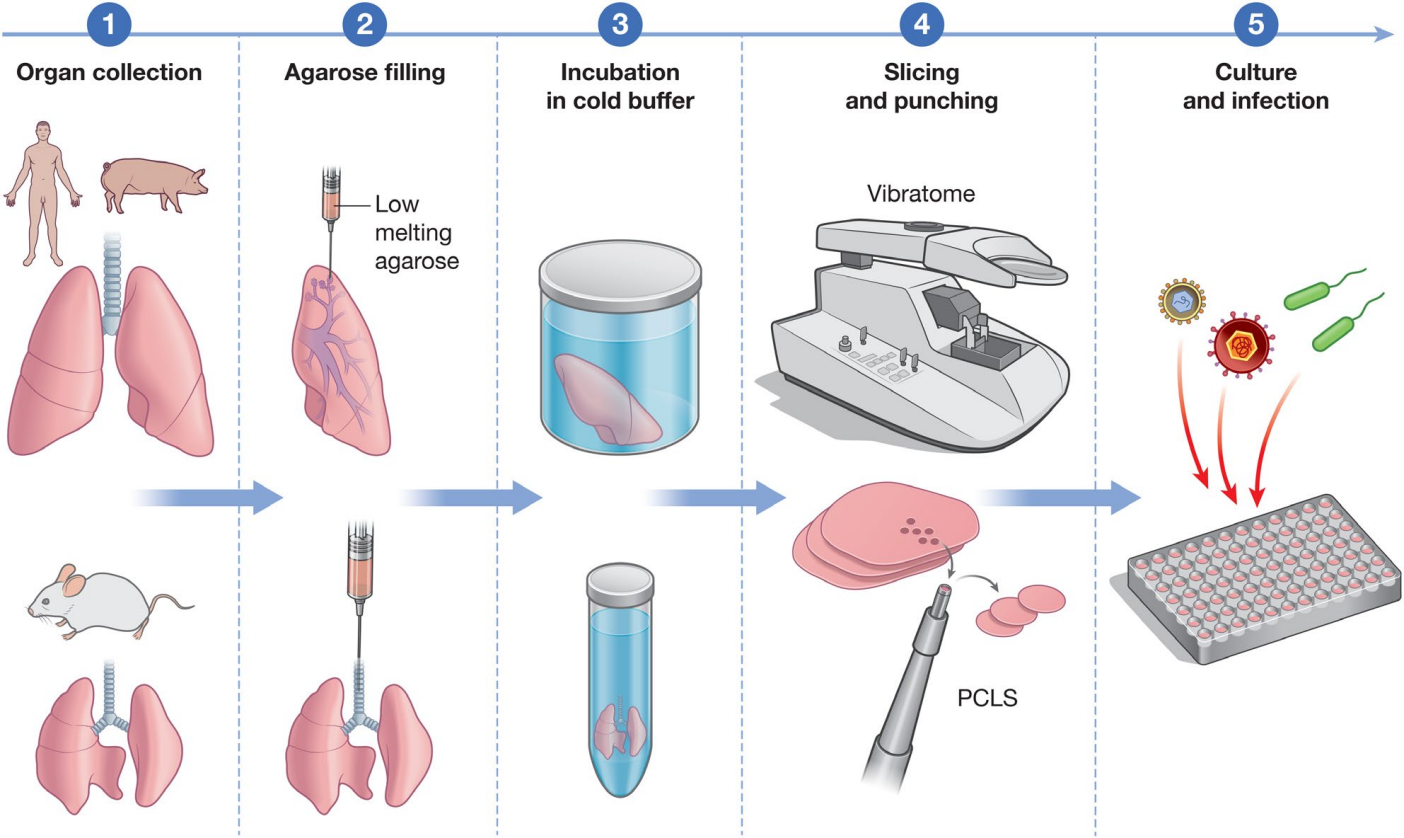
Generation of precision‐cut lung slices (PCLS) for infection studies. Scheme describing the key steps for generating PCLS from human and smaller animals lung tissue

Central to all protocols is that the freshly retrieved whole lungs or individual lobes are infused with warm low‐melting agarose dissolved in buffer or tissue culture medium, at a concentration ranging from 0.5% to 3% (Figure 1). The agarose can be administered through the trachea, for example for small animal lungs, or through smaller airways of dissected lobes. It is delivered at a constant slow rate and the total volume adjusted to the natural tissue volume to prevent tissue damage. Once the tissue is inflated, rapid solidification of the agarose is achieved by immersing the tissue in cold buffer, followed by incubation at 4℃. Upon solidification, the tissue is sliced using a vibrating microtome, yielding PCLS of uniform thickness between 150 and 500 µm. Thinner slices are typically obtained from smaller animals and thicker slices from larger animals and human tissue (Alsafadi et al., 2020). Variations of this procedure have been recently reviewed (Alsafadi et al., 2020). The PCLS are kept immersed in cell culture medium in well plates for downstream applications. Serum is either absent or added in very low quantities in order to prevent cell growth and changes to the native cellular diversity (Sanderson, 2011). The number of slices which can be generated is limited by the size of the organ, but a small human lung lobe can yield hundreds of PCLS (hPCLS). Uniform punches fitting 96 well plate wells can be generated from PCLS using biopsy punchers, facilitating high‐throughput applications. Reports on PCLS viability vary as this is likely affected by diverse factors (e.g., method for animal sacrifice, time, and conditions of conservation from tissue retrieval to slicing) (Sanderson, 2011) but hPCLS have been reported to remain viable for at least 15 days (Neuhaus et al., 2017).

## 3 PCLS: A NEAR NATIVE LUNG ENVIRONMENT TO STUDY AIRWAY BIOLOGY

The process of PCLS generation preserves most features of the native lung tissue, that is, its architecture and mechanical properties (Hiorns et al., 2016; Pybus et al., 2021) as well as a complex cellular composition, including resident lung cells, for example, fibroblasts, ATI and ATII epithelial cells, and immune cells, for example, macrophages, monocytes, and NK and T cells (Stegmayr et al., 2021). Neutrophils have been detected in murine PCLS (mPCLS) (Akram et al., 2019; Molina‐Torres et al., 2020) but considering that they are short‐lived ex vivo and that the PCLS are disconnected from blood and lymphatic circulation, their number is highly dependent on the way PCLS are generated and for how long slices are kept in culture. The absence of immune cell recruitment can be exploited to define the response of resident cells to infection and treatment.

**FIGURE 2:**
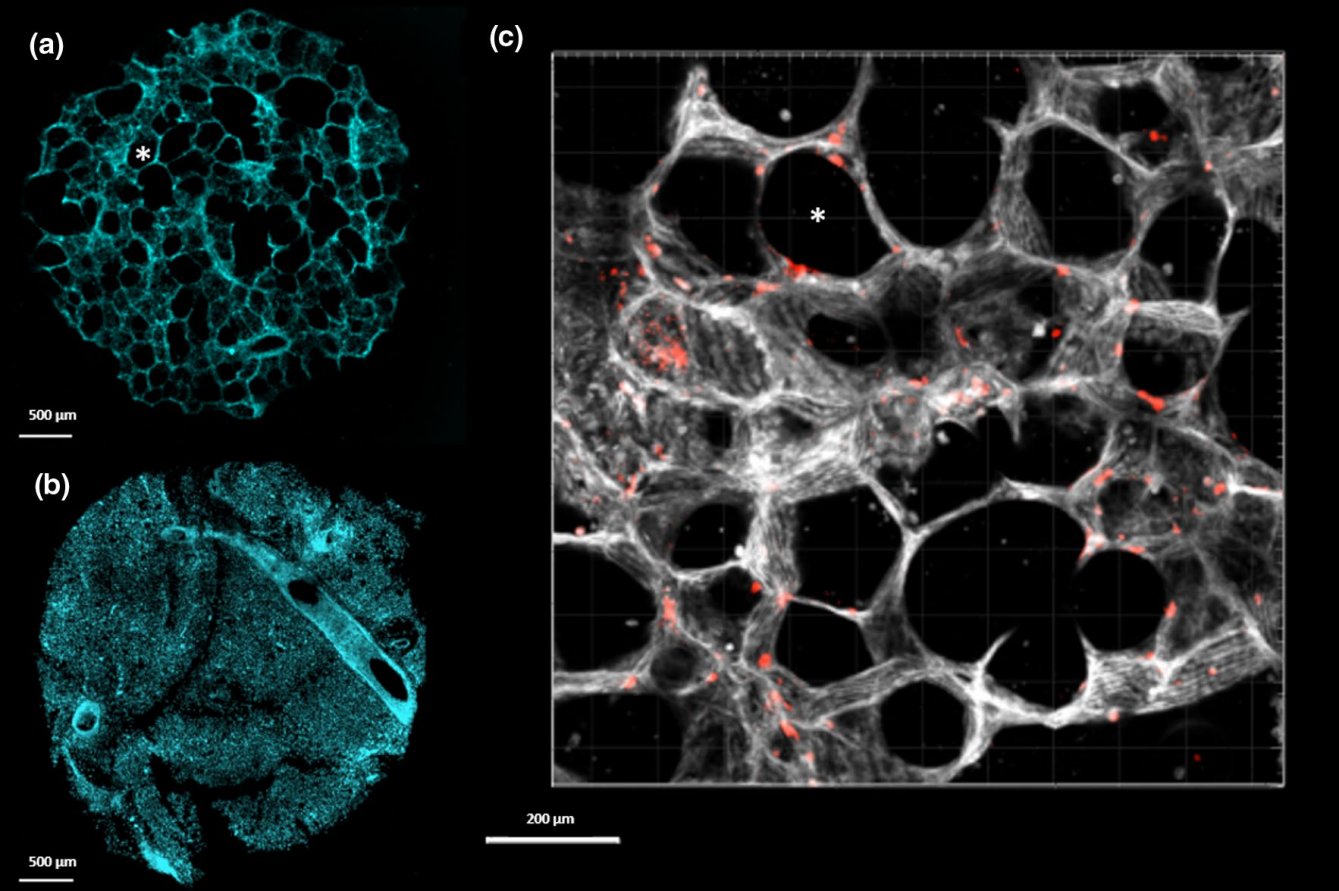
Comparison between human (a) and mouse (b) precision‐cut lung slices (PCLS) structure (Blue: DAPI staining of nuclei). (c) Section of an hPCLS infected with *Legionella pneumophila* (Red: expressing mScarlet‐I) 24 hr post infection (White: CellMask staining of cytoplasmic membranes). * mark alveolar spaces in (a) and (c). (a, c) were acquired by FV and (b) was acquired by FV and John Stegmayr

PCLS are ideally suited for high‐resolution live imaging, and ultrastructural changes, cell activity and migration, progression of infection, and drug treatments can be followed in real time using fluorescent markers and probes for different cell types, cell signaling or other processes, and/or fluorescently labeled pathogens (Figure 2). In parallel, cytokines, metabolites, or other molecules can be sampled from the PCLS culture medium. Like any other tissue, PCLS are also suitable for classical endpoint analysis by histology, immunohistochemistry, and immunofluorescence or transmission electron microscopy (Ebsen et al., 2002), metabolomics (Khan et al., 2020; Yilmaz et al., 2018), proteomics (Khan et al., 2020), and RNA isolation for transcriptome profiling (Niehof et al., 2017; Stegmayr et al., 2021).

Molecular mechanisms in PCLS can be probed with small molecule inhibitors, with the advantage that the amount of drug needed for well plate experiments is significantly reduced compared to in vivo studies, decreasing cost and facilitating assessment of drugs with limited availability. Moreover, genetic manipulation of PCLS by siRNA‐mediated protein knockdown has been achieved (Ruigrok et al., 2017, 2018). Notably, PCLS can also be obtained from healthy and diseased tissue from both human donors or animal models, as demonstrated for idiopathic pulmonary fibrosis (Alsafadi et al., 2017), chronic obstructive pulmonary disease (COPD), and asthma (Mertens et al., 2017), enabling the assessment of the interplay between these conditions and infections. Overall, these characteristics show that PCLS combine advantages of both in vitro and in vivo models and constitute an excellent ex vivo system to study lung biology, disease, and treatments. PCLS have therefore been widely adopted for pre‐clinical drug discovery and toxicological studies (Bäckström et al., 2016; Fisher et al., 1994; Hess et al., 2016; Lauenstein et al., 2014; Maihöfer et al., 2017; Neuhaus et al., 2017, 2018; Switalla, et al., 2010a) including the evaluation of immune modulators (Henjakovic et al., 2008; Leus et al., 2016; Switalla, et al., 2010b; Temann et al., 2017) and the induction of antigen‐specific T cell responses by new vaccine formulations (Neuhaus et al., 2013). Additionally, PCLS were also used to characterize tissue tropism and functional properties of gene therapy vectors and oncolytic viruses (Rosales Gerpe et al., 2018).

## 4 PCLS AS MODEL FOR HUMAN RESPIRATORY INFECTIOUS DISEASES

The first report of the use of PCLS to study the molecular mechanisms of infection focused on two respiratory pathogens, the Gram‐negative bacterium *C. pneumoniae* and paramyxovirus Respiratory Syncytial Virus (RSV) (Ebsen et al., 2002). *C. pneumoniae* is associated with wide range of diseases, for example, pulmonary emphysema, and RSV is the most common cause of viral infections in the lower respiratory tract of infants and children. Immunofluorescence and transmission electron microscopy revealed that both pathogens established infections and replicated in the mPCLS, evidenced by the characteristic chlamydial inclusions with spherical bodies of *C. pneumoniae* and RSV‐containing vacuoles in infected cells. Since this pioneering study, PCLS from different species have been used to investigate a wide range of human viral and bacterial pathogens. Here, we review these studies with emphasis on the different applications and readouts.

### 4.1 Viral pathogenesis in PCLS

#### 4.1.1 Paramyxoviruses

Paramyxoviruses are RNA viruses transmitted via inhalation of airborne droplets and able to cause diverse diseases in humans and animals. Apart from RSV, prominent members of this group are measles virus, the causative agent of measles, and metapneumovirus, an important cause of respiratory disease in particular in children (Park & Tishkowski, 2021; Troy & Bosco, 2016). PCLS from dogs, cynomolgus macaques, ferrets, and cotton rats, were assessed for their ability to support infection and discern differences in cell tropism and replication kinetics between the three human paramyxoviruses and canine distemper virus, the cause of distemper in carnivores (de Vries et al., 2018; Nguyen et al., 2013). Measles virus replicated optimally in macaque slices, metapneumovirus and RSV in cotton rat slices, and the canine distemper virus in slices from dogs and ferrets. This work not only established effective PCLS models for these viruses but, as PCLS were infected with recombinant virus expressing fluorescent proteins, also highlighted the potential of PCLS for real‐time monitoring of infection.

#### 4.1.2 Enteroviruses

Rhinoviruses (RVs) are the cause of the common cold, the most frequent human viral infection, and severe exacerbations of asthma and COPD (Troy & Bosco, 2016). Quantification of the RV load by quantitative PCR in hPCLS from asthmatic and healthy donors showed no differences between the groups at infection peak. However, RV triggered higher expression of cytokines, for example, *IL25, TSLP*, and *IL13*, in the tissue from asthmatics, indicating that RV‐induced exacerbations may be linked to an altered immune response (Kennedy et al., 2018). As hPCLS retain ciliary beating and are responsive to treatments with contractile stimuli such as the drug carbachol, the effect of RV infection on airway constriction was also analyzed, showing an enhanced contraction in infected asthmatic donor tissue (Parikh et al., 2020). Increased carbachol‐induced airway narrowing was also observed in mPCLS infected with rhinovirus C15 (RV‐C15). Both studies support a model in which altered immune responses in asthmatic airways following RV infection drive airway hyperresponsiveness.

PCLS derived from a mouse model of allergic asthma showed a compromised immune response to RV, that is, increased release of IL‐4, IL‐6, and IL‐10, which could be partially inhibited by the antiviral drug Rupintrivir (Danov et al., 2019). Bronchobini, a homeopathic multicomponent drug preparation marketed for inflammatory respiratory diseases, primed the antiviral host response in mPCLS suppressing excessive RV‐induced inflammation (Reamon‐Buettner et al., 2019). These studies illustrate the value of PCLS to study the effect of RV infection on airway immunology and contractility, and to discover new antiviral treatments.

#### 4.1.3 Adenoviruses

Adenoviruses cause diverse pathologies. Type 7 Adenovirus (Ad7) is associated with severe lower tract infection and pneumonia in both healthy and immunocompromised individuals, especially among children or adults living in crowded conditions (Clementi et al., 2021). Infection leads to infiltration of neutrophils in the lower respiratory tract and alveoli, followed by monocytes and later lymphocytes. Bovine and human PCLS support Ad7 replication and viral protein was detected in alveolar epithelial cells by immunohistochemistry (Booth et al., 2004). As in 2D models, PCLS infection triggered production and release of interleukin‐8 (IL‐8), the main chemoattractant for neutrophils, via the RAS/RAF‐I/MEKI/ERK pathway. A subsequent study in hPCLS, revealed that IL‐8 is mainly produced by type I alveolar epithelial cells, whereas IP‐10, a chemokine attracting monocytes and lymphocytes, is produced by epithelial cells and macrophages and seems to require communication of both cell types (Wu et al., 2010). These studies evidence the potential of PCLS to dissect cell tropism of pathogens and intercellular communication in the host response.

#### 4.1.4 Influenza viruses

Influenza A virus is a major cause of global morbidity and mortality. Annual flu epidemics claim tens of thousands of lives and intermittent pandemics have an even higher death toll (Troy & Bosco, 2016; World Health Organization (WHO), 2018). Rapid evolution of the virus creates the need for effective model systems for the characterization of new isolates and the development of treatments. Liu et al. optimized mPCLS for quantifying the replication of human influenza A strains PR8 (H1N1) and HUBEI (H3N2) by viral titer measurement and a fluorometric neuraminidase (NA) activity assay (Liu et al., 2015). Moreover, mPCLS and bronchoalveolar lavage fluid (BALF) from infected mice displayed a similar increase of cytokines and chemokines, such as IP‐10, RANTES and MIP‐3α, and a similar response to a number of antiviral and anti‐inflammatory agents (ribavirin, oseltamivir, germacrone, U0126, EGCG, 15d‐PGJ2, and SB203580). Overall, this validated mPCLS as a predictive, time and cost‐economic model for studying pathogenesis, and treatment of human influenza A virus.

The mPCLS model also enables the assessment of complex interactions of infection, drug treatments, and environmental factors, such as smoking, a main risk factor for COPD. Influenza A virus is a known driver of exacerbations of COPD and asthma. PCLS from smoke‐exposed mice revealed that smoke had no effect on clearance of H1N1 virus, but was associated with higher expression of inflammatory chemokines such as MCP‐1/‐3, KC, MIP‐2, and GCP‐2 (Bauer et al., 2010). The same model showed that smoke alone and in combination with Influenza A virus infection (Mem71, H3N1) impaired β‐adrenoceptor sensitivity and responsiveness of small airways to the β2‐adrenoceptor agonist bronchodilator salbutamol (SALB) (Donovan et al., 2016).

PCLS from marmosets and rhesus and cynomolgus macaques were also validated as Influenza A (Hamburg/04/2009 (H1N1) or A/PR/8/34 (H1N1)) infection models and confirmed that the virus exploits in all models the activity of the protease TMPRSS2, which cleaves and activates the viral hemagglutinin (HA) promoting replication and spread (Zmora et al., 2017).

While not always reproducing all human disease features, animal PCLS also offer an effective system to assess the virulence of different isolates and their potential for interspecies transmission. The most severe Influenza A pandemics are often driven by virus subtypes, which recently breached the species boundary from animals to humans. A good correlation of the virulence of swine influenza viruses in vivo and replicative capacity and ciliostatic effect in porcine PCLS (pPCLS) was observed, showing that PCLS models deliver meaningful results for the comparison of the pathogenicity of different isolates (Meng et al., 2013). In pPCLS, swine influenza A virus (H3N2) replicated better than two avian influenza viruses (H9N2 and H7N7), but H9N2 replicated robustly in lung epithelial cells, indicating potential for interspecies transmission (Punyadarsaniya et al., 2011). Adaptive evolution experiments in pPCLS showed that after three passages avian influenza virus H9N2 evolved its HA protein to bind α2,6‐linked sialic acids, typical surface receptors for swine and human viruses, in addition to the α2,3‐linked sialic acids commonly used by avian subtypes (Yang et al., 2017). These mutants displayed enhanced replication in mice but not in pPCLS, suggesting that other adaptive mutations might still be required for virulence in pigs. These studies demonstrate the usefulness of PCLS to model zoonotic infections and assess the risk for emergence of new human pathogens.

#### 4.1.5 Coronaviruses

The SARS‐CoV‐2 pandemic generated the urgent need for relevant preclinical drug discovery platforms. hPCLS were employed for the characterization of the antiviral effect of Camostat mesylate, an inhibitor of TMPRSS2 and related proteases, which are required for proteolytic activation of the viral S protein and cell invasion (Hoffmann et al., 2021), and in a drug repurposing screen for inhibitors of SARS‐CoV‐2 replication (Zimniak et al., 2021). SARS‐CoV‐2 replicated in hPCLS as measured by quantitative RT‐PCR and viral titer. Camostat mesylate and Fluoxetine, a selective serotonin reuptake inhibitor used for treatment of depression, reduced viral burden. Notably, Lopinavir, which repressed viral replication in in vitro 2D infection models, failed to reduce the viral titers in hPCLS. This shows the value of this more complex, ex vivo tissue‐based infection model for the effective discovery of antimicrobials and for studying the pathogenesis of SARS‐CoV‐2 in the future.

### 4.2 Bacterial pathogenesis in PCLS

Since the first ever use of PCLS as a model for respiratory infection with *C. pneumoniae* (Ebsen et al., 2002), an increasing number of studies have exploited PCLS to investigate how bacterial pathogens cause disease in humans. Additional studies on zoonotic pathogens, for example, *Bordetella bronchiseptica*, that focus on aspects of pathogenesis in the natural animal hosts have been discussed elsewhere (Vötsch et al., 2021).

#### 4.2.1 Staphylococcus aureus

*Staphylococcus aureus* is a multifaceted Gram‐positive pathogen of high concern due to increasing multidrug resistance. Methicillin‐resistant *S. aureus* (MRSA) and Methicillin‐sensitive strains (MSSA) cause community‐pneumonia in healthy individuals, but MSSA are associated with a lower mortality rate. As murine macrophages were found to be growth permissive, it was speculated that macrophages modulate pathogenesis in humans. However, both *S. aureus* types survive, but do not replicate in isolated human alveolar macrophages, and in hPCLS the bacteria are mostly found in *S. aureus*‐containing phagosomes in epithelial cells and interstitial regions (Brann et al., 2019). Only limited numbers are detected in alveolar macrophages. Thus, human alveolar macrophages are most likely not a key permissive replicative niche, pointing to other cell types as drivers of *S. aureus* lung infection. This study highlights the potential of PCLS to investigate cell tropism and key cellular interactions of bacterial pathogens.

#### 4.2.2 Coxiella burnetii

*Coxiella burnetii* is an obligate intracellular pathogen that causes Q‐fever in humans, which can progress from flu‐like symptoms into a life‐threatening endocarditis (Dragan & Voth, 2020). In cell culture models, the bacteria replicate inside acidic parasitophorous vacuoles (PVs) in human alveolar macrophages and also non‐phagocytic cells. hPCLS infection established that while individual bacteria could be detected in different cell types, replication occurred in PVs in alveolar macrophages (Dragan et al., 2019; Graham et al., 2016). Replication in PCLS depended on the Dot/Icm type 4 secretion system, which is essential to deliver effector proteins for manipulation of host processes and biogenesis of the PV. No substantial structural damage to the PCLS tissue was observed even after 72 hr of infection. Infection induced the production and caspase‐1‐dependent release of mature IL‐1β from PCLS. Notably, these experiments were performed with the attenuated, laboratory strain *C. burnetii* NMII (RSA439, avirulent clone 4) (Millar et al., 2017), which induces a markedly different inflammatory response than wild type bacteria. Nevertheless, this study highlighted how hPCLS represent a promising model to investigate the *C. burnetii* pathogenesis in a physiological context.

#### 4.2.3 Bacillus anthracis

*Bacillus anthracis* is a spore‐forming Gram‐positive pathogen. Pulmonary infection with *B. anthracis* spores causes inhalation anthrax, the most severe and deadly form of the disease. Early innate immune responses to *B. anthracis* spores were investigated in hPCLS, showing Mitogen‐Activated Protein Kinase (MAPK)‐driven proinflammatory cytokine and chemokine production by macrophages and lung epithelia, including IL‐8, MCP‐1 and MIP‐1α/β, potent chemoattractants for neutrophils, and monocytes (Chakrabarty et al., 2007). This was the first evidence of an active involvement of the lung epithelium in the innate immune response to *B. anthracis* spores and contrasts with the lack of cytokine production observed in a complex 3D cell model of primary small airway epithelial cells cultured in collagen matrices in presence of peripheral blood monocytes (Radyuk et al., 2003). While hPCLS likely represent a more accurate lung model and thus show more realistic early innate immune responses, this awaits validation by autopsy data.

#### 4.2.4 Mycobacteria

Mycobacterial infections are a global health problem (Gopalaswamy et al., 2020). While *Mycobacterium tuberculosis* (MTb) remains the leading cause of death by a single infectious agent, several species of non‐tuberculous mycobacteria (NTM) are emerging as important cause of opportunistic infections. Treatment requires multidrug therapy over several months and is associated with high failure rates. This is due to increasing multi‐drug resistance of MTb and high intrinsic antibiotic resistance of NTMs. Moreover, phenotypic heterogeneity of the bacteria in the host, for example, formation of nonreplicating persisters resistant to antibiotics, promotes infection relapses. As in vitro drug screening assays do not reflect these conditions well, PCLS have been evaluated as models for anti‐mycobacterial drug discovery.

*Mycobacterium abscessus* (MAb) is a fast growing NTM and imminent multidrug‐resistant health threat for patients with respiratory conditions such as COPD and cystic fibrosis (CF) (Molina‐Torres et al., 2020). Upon infection of mPCLS, progressive tissue damage occurred, for example, alveolar edema, vascular congestion, and extravasion of lymphocytes and erythrocytes in the septa and alveolar spaces with rupture and thickening of alveolar septa by 48 hr post infection, concomitantly with infiltration of histiocytes, aggregates of foamy macrophages, and fragmentation of polymorphonuclear cells (Molina‐Torres et al., 2020). MAb were found in macrophages and type I and II pneumocytes. Proof‐of principle antibiotic treatment assays with imipenem and tigecycline demonstrated that tigecycline lead to a significant reduction of bacterial burden in a dose‐ and time‐dependent manner while imipenem induced only a moderate reduction.

MTb is a slowly growing bacterium causing mostly chronic disease. hPCLS were employed to study infection and the immune response within the first 24 hr of MTb infection in comparison with *Mycobacterium bovis* BCG (Carranza‐Rosales et al., 2017). Bacteria were found associated with alveolar septa, alveolar light spaces, near type II pneumocytes, and resided in macrophages. Immune cells were recruited to sites where bacteria accumulated, showing that PCLS retain some of the cellular immune responses of the native tissue. No significant change of bacterial load was observed over time and infection triggered the production of TNF‐α, mirroring findings in other in vitro and in vivo studies.

Overall, these two studies show that PCLS can be a useful tool for fundamental mycobacterial research and drug discovery.

#### 4.2.5 Pseudomonas aeruginosa

*Pseudomonas aeruginosa* is a versatile opportunistic pathogen causing acute and chronic respiratory infections, particularly in immunocompromised, COPD, and CF patients. The bacteria use different virulence factors including a flagellum and type 2 and type 3 secretion systems to secrete diverse toxins and enzymes (Jurado‐Martín et al., 2021). However, how both these virulence factors and pathogen‐associated molecular patterns such as LPS, drive the immune response in the human lung is not fully understood. Using mPCLS exposed to different live and heat‐killed clinical isolates, Kolbe et al. showed that infection with live but not dead bacteria results in substantial transcriptional reprogramming involving increased cytokine expression (Kolbe et al., 2020). Comparison of bacterial mutants in combination with pharmacological inhibition of phagocytosis, showed that this response to viable bacteria required their internalization and depended on the detection of flagellin and the T3SS. Moreover, employing PCLS from knock‐out (KO) mice, the redundant involvement of the receptors MARCO and CD200R1 in bacterial uptake was also demonstrated. This illustrates that PCLS enable parallel assessment of multiple bacterial strains in the near‐native lung tissue from the same animal. Additionally, in combination with inhibitors and KO animals, PCLS provide further insight in the molecular mechanisms of host‐pathogen interactions.

#### 4.2.6 Yersinia pestis

*Yersinia pestis* is the causative agent of plague. Inhalation of *Y. pestis* leads to pneumonic plague, which can be fatal within days if untreated. Virulence factors, for example, a type 3 secretion system that injects effectors for host manipulation and the protease Plasminogen activator (Pla), are essential for *Y. pestis* infection and replication, as well as for dissemination and disease progression in cellular and animal models (Demeure et al., 2019). The exact function of Pla however remained an enigma. An hPCLS model of pneumonic plague combined with the fluorescence‐based TEM1‐β‐lactamase effector translocation assay revealed that Pla promotes optimal translocation of effectors into phagocytes, especially alveolar macrophages (Banerjee et al., 2019). In contrast, Pla was dispensable for type 3 secretion into THP‐1 cells, an immortalized cell line considered as macrophage surrogate model. Moreover, in the PCLS infection model, Pla plays a vital role for the inhibition of the expression of the proinflammatory cytokines IL‐6, IL‐8, and TNFα during the first hours of infection. During this phase, it is not possible to measure cytokine levels in mouse lungs as these are below detection level. This reinforces the notion that the functions of virulence factors might only become apparent under physiological infection conditions and that PCLS are a unique system to investigate these functions and early events of infection, which are usually poorly accessible.

## 5 CONCLUSION & OUTLOOK

Bridging the gap between in vitro and in vivo models, PCLS are a reliable and powerful model to investigate respiratory infectious diseases. hPCLS in particular deliver highly relevant data reflecting human disease, eliminating the often speculative extrapolation of findings from surrogate models to humans. As many human pathogens are often unable to infect and replicate in certain animal models, hPCLS can also provide an important platform for modeling viral and bacterial co‐infections, abrogating the host‐specificity issues that hinder such studies. Some constraints remain, for example, limited genetic tractability, lack of cell infiltration, and of adaptive immunity, as well as the short life span of the slices which hinders their use for studying chronic infections, but these limitations are being actively tackled, for example, by embedding PCLS in hydrogels that expand their life span (Bailey et al., 2020). Given the exceptional accessibility of PCLS for live imaging and the option to compare many pathogen strains or treatments, PCLS promise to become an invaluable tool for dissecting the molecular mechanisms of host‐pathogen interactions within the physiological context of lung tissue and cellular microbiology research in the future.

## CONFLICT OF INTEREST

FV and GNS declare that they have no conflicts of interest with the contents of this article. COK has received consultancy fees from Insmed for the treatment of pulmonary non tuberculous mycobacterial infection.

## Data Availability

Data sharing is not applicable to this literature review as no new data were generated. Micrographs in Figure 2 are merely included for illustrative purposes.
